# Confronting barriers to human-robot cooperation: balancing efficiency and risk in machine behavior

**DOI:** 10.1016/j.isci.2020.101963

**Published:** 2020-12-17

**Authors:** Tim Whiting, Alvika Gautam, Jacob Tye, Michael Simmons, Jordan Henstrom, Mayada Oudah, Jacob W. Crandall

**Affiliations:** 1 Brigham Young University, Provo, UT 84602, USA; 2 Oregon State University, Corvallis, OR 97331, USA; 3 New York University Abu Dhabi, Abu Dhabi, UAE

**Keywords:** Human-Computer Interaction, Social Sciences, Psychology

## Abstract

Many technical and psychological challenges make it difficult to design machines that effectively cooperate with people. To better understand these challenges, we conducted a series of studies investigating human-human, robot-robot, and human-robot cooperation in a strategically rich resource-sharing scenario, which required players to balance efficiency, fairness, and risk. In these studies, both human-human and robot-robot dyads typically learned efficient and risky cooperative solutions when they could communicate. In the absence of communication, robot dyads still often learned the same efficient solution, but human dyads achieved a less efficient (less risky) form of cooperation. This difference in how people and machines treat risk appeared to discourage human-robot cooperation, as human-robot dyads frequently failed to cooperate without communication. These results indicate that machine behavior should better align with human behavior, promoting efficiency while simultaneously considering human tendencies toward risk and fairness.

## Highlights

• Experiments show that people learned risk-averse solutions without communication
• With and without communication, robot pairs learned risky, but efficient, outcomes
• Human-robot pairs often learned risky, but efficient, solutions with communication
• Without communication, behavioral asymmetries inhibited human-robot cooperation

## Graphical Abstract

**Figure:**
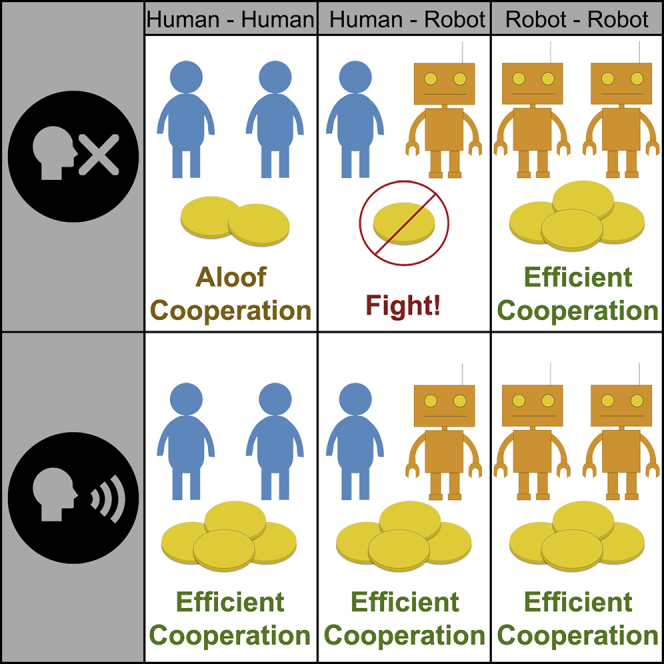

## Introduction

Smart devices, assistive technologies, medical assistants, recommender systems, driverless cars, and many other automated systems are increasingly permeating the human society. These systems are being endowed with increasingly sophisticated machine learning algorithms and other forms of artificial intelligence so that they behave in ways that match and even exceed human capabilities. As these automated systems engage with people in important tasks, including safety-critical ones, care must be taken to ensure effective cooperation and coordination with people, especially when the preferences given to the machine do not fully align with the preferences of their human partners.

When human and machine preferences are not fully aligned, machines must overcome a variety of barriers to facilitate human-machine cooperation. We broadly group these barriers into two categories. The first category relates to the attitudes people display toward machines that impact cooperation. For example, prior work has shown that people are more likely to lie and cheat when they believe they are interacting with a machine rather than a human (Kiesler et al., 1996; Cohn et al., 2018), offer less incentives to potential machine partners than potential human partners when negotiating the formation of teams (van Wissen et al., 2012), and are less inclined to cooperate with machine partners than human partners in prisoner's dilemmas (Ishowo-Oloko et al., 2019). Importantly, a recent study revealed that people fail to activate *mentalizing regions* of the brain when they believe they are interacting with an artificial agent (as opposed to another person) (Chaminade et al., 2012). As in human-human interactions (Sally, 1995; Balliet, 2009), these attitudes can potentially be overcome or influenced using, among other things, communication (e.g., Crandall et al., 2018) and physical appearance (e.g., Goetz et al., 2003), although thoroughly addressing these and other related barriers to human-machine cooperation remains an open research question.

The second set of barriers to human-machine cooperation is related to the strategic behavior of machines (Rahwan et al., 2019). When the preferences of individuals are not fully aligned, the effectiveness of a strategy often depends on the behavior of one's associates (for example, see Axelrod's discussion about iterated prisoner's dilemmas, Axelrod, 1984). This results in highly complex solution spaces, which makes computing and determining effective strategies difficult (e.g., Daskalakis et al., 2009; Gintis, 2000). The methods most commonly used by machines to compute or learn behavior are based on gradient ascent or other related optimization techniques. These methods can produce effective behavior in zero-sum games, including Checkers (Schaeffer et al., 2007), Chess (Campbell et al., 2002), Poker (Bowling et al., 2015; Moravčík et al., 2017), and Go Silver et al. (2016). However, in many other scenarios (including social dilemmas and repeated general-sum games) in which cooperation is beneficial but nontrivial, these algorithms often produce myopic, non-cooperative, and low performing (Crandall et al., 2018) behavior due to the non-stationary dynamics of the environment (caused by the different players adapting to each other) and the multiplicity of available equilibria.

Substantial effort has been dedicated to developing modified or alternative methods for (repeated) general-sum games. These efforts have allowed machines to learn strategies that induce cooperation (with varying levels of success) in some contexts (e.g., Sandholm and Crites (1996); Littman (2001); Powers and Shoham (2005); Littman and Stone (2005, 2001); De Cote and Littman (2008); Gal et al. (2010); Albrecht and Ramamoorthy (2013); Elidrisi et al. (2014); Crandall (2014); Leibo et al., 2017; Foerster et al. (2018)). For the studies conducted in this article, we equipped our robot with S#, a multi-agent learning algorithm that has, in prior work, demonstrated the ability to induce human-machine cooperation in repeated games under certain communication conditions (Crandall et al., 2018).

Although S# has demonstrated the ability to establish cooperative relationships with people in a variety of repeated games when it can communicate, it often fails to establish cooperative relationships with people when communication is not possible (Crandall et al., 2018). To better understand why S# sometimes fails to establish human-machine cooperation, we conducted a series of user studies in the *Block Dilemma* (Oudah et al., 2015), a repeated game in which a suite of cooperative solutions that differ with respect to efficiency, risk, and fairness is available to the players. The results from these user studies suggest that S#'s failure to establish cooperative relationships with people under certain circumstances is, to some degree, due to differences in how S# and people approach the efficiency, risk, and fairness of solutions. S# (along with many other artificial intelligence [AI] algorithms) focuses almost exclusively on learning and promoting efficient solutions that give the algorithm high expected payoffs, whereas people attune their behavior to risk (Kahneman and Tversky, 1979) and fairness (Grgić-Hlača et al., 2018; Schweitzer and Gibson, 2008; Joseph and Willis, 1963; Pruitt and Carnevale, 1993). This misalignment in behavior sometimes causes conflict between people and machines that inhibits cooperation and coordination, which indicates that machines whose behaviors address human inclinations toward risk and fairness are more likely to elicit human-machine cooperation and coordination than those that do not.

## Results

**Figure 1:**
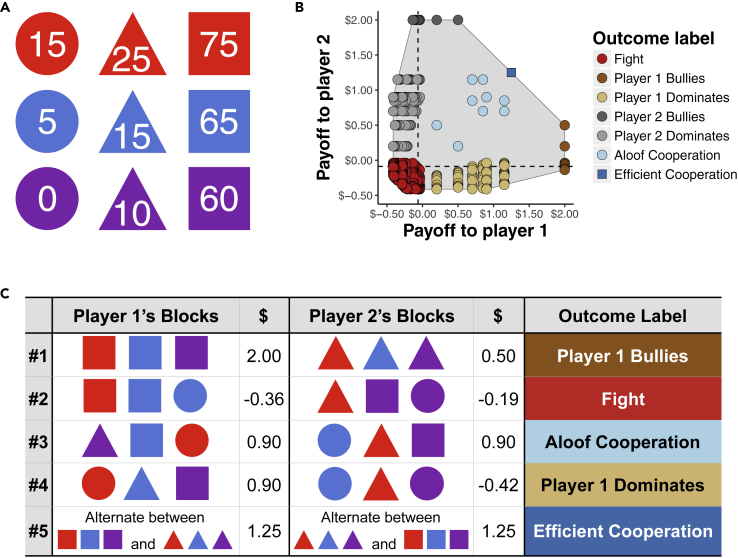
An overview of the Block Dilemma (A) In each round of the Block Dilemma, the players take turns selecting blocks from this set of nine blocks until each player has three blocks. Numbers indicate the monetary value (in cents USD) of each block. (B) The joint payoff space of a single round. The gray-shaded polygon shows the convex hull of the joint payoff space. Dashed lines indicate the players' maximin values. Each circle represents a possible joint payoff of a single round. The dark blue square (Efficient Cooperation) shows the average joint payoff that results from the players taking turns getting the squares and triangles across consecutive rounds. The outcomes are grouped (by color) and labeled for ease of exposition. (C) Five typical round outcomes, the associated (average) per-round value (in USD) of the outcomes to the players, and the labels we use to categorize the outcomes.

To better understand when and how human-machine cooperation emerges, we conducted a series of simulations and user studies in the Block Dilemma (Oudah et al., 2015), which is overviewed in Figure 1. In the Block Dilemma, two players interact in a series of rounds. In each round, the players play an extensive-form game in which they take turns selecting blocks from the set of nine blocks shown in Figure 1A, with player 1 always selecting a block first in each round. A round ends when each player has selected three blocks. The number on each block indicates its monetary value in cents (USD). When a player's three blocks form a valid set (i.e., all their blocks have the same color, or have the same shape, or have nothing in common), then the player's earnings in the round are the sum of the numbers on their blocks. The round earnings of a player who fails to collect a valid set of blocks is the sum of the numbers on their blocks divided by −4, meaning that a player who does not get a valid set loses money in that round.

Figures 1B and 1C illustrate the set of potential outcomes of each round of the Block Dilemma. The game includes unfair solutions, which are substantially more beneficial to one player than the other; fair outcomes in which both players receive relatively high payoffs (outcomes we refer to as *Aloof Cooperation*, as they require the players to coordinate behavior but not interact extensively); and outcomes in which both players lose money. Another efficient and fair (yet risky) outcome is achieved over multiple rounds when the players take turns getting the squares and triangles. We call this solution *Efficient Cooperation*, because it provides both players with higher average earnings than *Aloof Cooperation* (which is less risky). A more detailed description and analysis of the Block Dilemma is given in the Supplemental information (see Transparent methods: SM 1).

In our first study, we observed the behavior of human-human pairs in the Block Dilemma. In study 2, we consider the behavior of pairs of robot in this game. These first two studies provide context for the behavior of human-robot dyads, which we analyze in studies 3 and 4.

### Study 1: human behavior in the Block Dilemma

In the first study, each pair of participants was assigned to one of two conditions: *unrestricted communication* or *no communication*. Figure S1 shows the physical setup for these two conditions. Pairs of players assigned to the condition with unrestricted communication were allowed to freely communicate with each other in any way they pleased, both verbally and non-verbally. Pairs assigned to the *no communication* condition were not allowed to communicate with each other in any way. To help keep the players from communicating (including nonverbal communication), we attached blinders to the table, which allowed the players to see the blocks on the table but not each other's faces. Participants in this condition were instructed to not attempt to use any verbal or non-verbal communication during the game; experiment administrators further ensured that they did not do so.

Forty people (20 pairs) participated in this study. Each interaction lasted 15 rounds, although participants were not told in advance how many rounds their interaction would last. Participants were paid the amount of money that they earned throughout the 15 rounds of the game they played. Additional details about the study design are given in the Supplemental information (see Transparent methods: SM 2).

**Figure 2:**
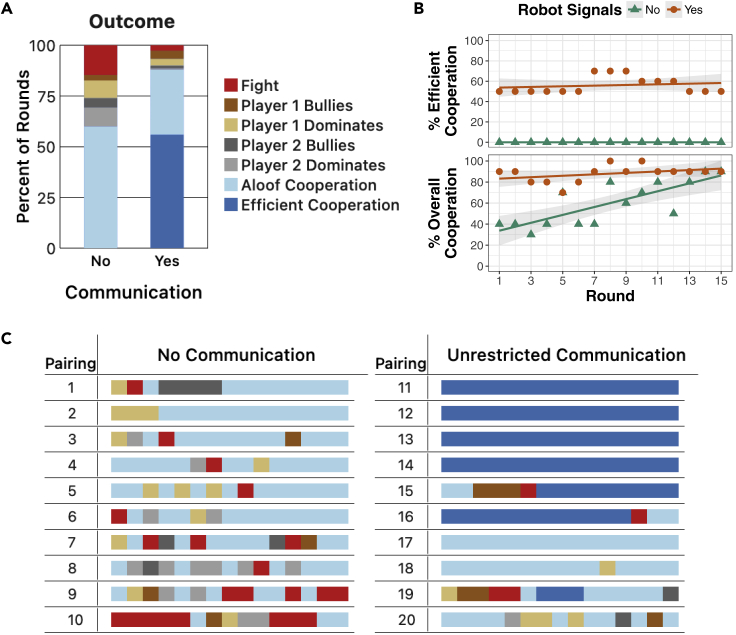
Results of human-human pairings in the Block Dilemma (A) The percentage of rounds across all pairings that resulted in each category of round outcome. (B) Cooperation rates per round over all pairings, with the top plot showing the percentage of interactions resulting in *Efficient Cooperation* and the bottom plot showing the percentage of interactions resulting in either *Efficient* or *Aloof Cooperation*. For better visualization, fitted lines show a linear model of the data, with shaded areas representing 95% confidence intervals of the model's fit. (C) The outcome of each of the 15 rounds of each pairing. For example, in Pairing #2 (no communication), player 1 dominated player 2 in the first three rounds, followed by *Aloof Cooperation* in the remaining 12 rounds. In Pairings #11–14 (unrestricted communication), the players efficiently cooperated (by taking turns getting the squares) through all 15 rounds of the interactions.

The results of the study are summarized in Figure 2. In both conditions (with and without communication), most rounds resulted in cooperation, with bullying behavior being rather rare (Figures 2A and 2C). However, when people were not allowed to communicate with each other, *Aloof Cooperation* rather than *Efficient Cooperation* was prevalent. In fact, across all 10 pairings in which communication was not permitted, there was no instance of *Efficient Cooperation* (in which the players took turns getting the squares in consecutive rounds). Thus, whereas people mostly refrained from fighting and bullying in these pairings and generally cooperated with each other (with cooperation becoming more prevalent over time; Figure 2B), they did not attain *Efficient Cooperation* when they could not communicate.

Consistent with past research on the impact of communication on cooperation (Pentland, 2010; De Melo et al., 2009; de Melo et al., 2014; Oudah et al., 2015, Oudah et al., 2018; Correia et al., 2018; Breazeal et al., 2005; Oliveira et al., 2018; Taheri et al., 2014; Ricks and Colton, 2010), cooperation among people was higher when participants were allowed to communicate with each other. A Mann-Whitney test indicates that the number of rounds of cooperation in an interaction was greater under unrestricted communication than under no communication (U=14; p=0.007). Furthermore, unlike the condition without communication, pairs of people frequently converged to *Efficient Cooperation* given the ability to communicate, often beginning in the first round of the interaction (Figures 2B and 2C). As a result of both more frequent cooperation and more profitable cooperation, participants who could communicate with each other earned more money than did participants without communication. Participants in the unrestricted-communication condition earned $1.08 per round, whereas those in the no-communication condition earned just $0.67 per round.

Why did pairs of participants opt for the less efficient (although less risky) outcome without communication? An analysis of the communication of participants who were allowed to communicate with each other provides some insights. First, communication was used by participants to *discover Efficient Cooperation* as a desirable solution. Most pairs of participants who could communicate discussed which round outcome was ideal. For some pairs, the desirability of *Efficient Cooperation* over *Aloof Cooperation* was immediately obvious to both players. To other pairs, it was not so obvious, but communication helped the players to sort it out. For example, in Pairing #12, whereas player 2 suggested alternating between sets of squares and triangles starting in the first round, player 1 initially advocated for alternating between sets of red and blue blocks. After further discussion, they agreed to take turns getting the squares. That said, communication did not help all pairs of players in this way. In Pairing #17, for example, player 1 advocated before the first round that taking turns getting sets of red and blue blocks was ideal. Player 2 agreed and conformed throughout the interaction without verifying the calculation.

In addition to helping the players discover which solution to play, communication also helped many pairs of players negotiate and coordinate *Efficient Cooperation*. As a prime example, we consider Pairing #15. After the first round, the players began discussing how they could both “win.” However, it was not until after the second round that they deliberately calculated and discussed that taking turns getting the squares and triangles would be better than other forms of cooperation. Despite discussing this form of cooperation, for the next three rounds player 1 took the squares each round (and player 2 went along with it). During these rounds, player 1 theorized that he could bully his partner and always get the squares because he selected the blocks first. Toward the end of round 5, player 2 speculated (in a friendly way) about whether or not retaliation would be a good strategy against such bullying, and in round 6 decided to carry it out (which led to neither player getting a valid set that round). Following this verbal threat and then the carrying out of this threat, player 1 allowed player 2 to get all the squares in round 7, and the players alternated between getting squares and triangles for the remainder of their interaction.

In summary, communication helped human players to discover efficient solutions, to negotiate which solutions to play, and to coordinate their behavior. Together, these benefits of communication allowed many pairs of human players to frequently achieve *Efficient Cooperation*. On the other hand, none of the pairs of players in the no-communication condition were able to do so. Without communication, neither player can easily confirm that the other player thinks alternating between squares and triangles is a good idea nor can they easily coordinate who should get the squares first. Additionally, without communication, a player cannot easily confirm whether allowing the other person to have the squares will be reciprocated in the next round or whether allowing the other player to get the squares will be viewed as weakness. Finally, without communication, a player cannot communicate the threat that if the other player does not conform with efficient cooperation, he or she will keep the other player from getting a valid set. Together, these barriers either discouraged the players from attempting to play this more efficient (albeit more risky) form of cooperation, or made such attempts by individual players ineffective.

### Study 2: machine behavior in the Block Dilemma

As a point of comparison, we now consider interactions between machines in the Block Dilemma. Over the last several decades, many different algorithms have been developed for playing repeated games (e.g., Littman (1994); Hu and Wellman (1998); Littman (2001); Bowling and Veloso (2002); De Cote and Littman (2008); Johanson et al. (2012); Albrecht and Ramamoorthy (2013); Crandall (2015); Silver et al. (2016)). In this article, we chose to use the multi-agent learning algorithm S# (Crandall et al., 2018) to control our robot's strategic behavior due to (1) its high and robust performance demonstrated in prior evaluations (including in the Block Dilemma, Oudah et al., 2015), (2) its ability to adapt quickly (a requirement for the relatively brief 15-round interactions we consider in this research), and (3) its ability to utilize communication signals when interacting with people.

S# is an extension of S++ (Crandall, 2014), an expert algorithm that learns through experience which strategy to follow from among a set of computed strategies $E=\left\{e_{1},\cdots ,e_{k}\right\}$. The set *E* includes (among others) the best response strategy given the empirical distribution of its partner's past behavior (which typically converges to *Aloof Cooperation*), the agent's maximin strategy, and a variety of Pareto-optimal compromises and trigger strategies (Gintis, 2000). In the Block Dilemma, these Pareto-optimal compromises (which are paired with threats to punish in case of deviations from the compromises) include bullying (insisting on always getting the squares), *Efficient Cooperation* (taking turns getting the squares and triangles), and being bullied (allowing one's partner to always get the squares).

In addition to computing the set *E* from a description of the game and the history of play, S++ computes in each round *t* the potential of each strategy $e_{j}\in E$ (denoted $\rho_{j}\left(t\right)$) and its aspiration level α(t), which encodes the average per-round payoff the agent should expect to receive if it is successful. Based on these potentials and the aspiration level, S++ computes the set

(Equation 1) $$E\left(t\right)=\left\{e_{j}\in E:\rho_{j}\left(t\right)\geq \alpha \left(t\right)\right\}.$$

In words, E(t) consists of the strategies the agent believes could meet its aspirations should its partner conform. In each round, S++ selects an expert strategy from the set E(t) and follows that strategy throughout the round. Over time, it learns which expert strategy to follow by adjusting its aspiration level α(t) toward its average payoffs (which in turn varies the set E(t)) and identifying whether its currently selected strategy actually achieves its aspirations.

The set E(t) (as defined in Equation 1) prioritizes the potential efficiency of strategies rather than the risk of solutions. Strategies that are perceived to not have the potential to produce sufficiently high payoffs are excluded from E(t). When the aspiration level α(t) is initially set high, the agent will only select potentially efficient solutions. Only after these potentially efficient solutions have proven unsuccessful (which will result in decreases in the aspiration level α(t) over time) will less efficient solutions be considered by the agent. This mechanism has been shown to be responsible for the fast and effective learning behavior of S++ in repeated games when compared with other expert algorithms (Crandall, 2014), as well as its ability to quickly learn cooperative behavior when associating with people (Crandall et al., 2018). Importantly, in the Block Dilemma, this mechanism causes the algorithm in early rounds of the interaction to not consider *Aloof Cooperation*, but to instead seek to either bully the other player or to play *Efficient Cooperation*. The exception to this rule is that our implementation of S++ by default selects its best-response strategy in round 1, which can result in *Aloof Cooperation* in rounds 1 and 2..

S# is identical to S++ when communication between players is not permitted. However, when communication is possible, S# utilizes speech to negotiate a desirable solution with its partner. It does so by (1) voicing the strategy it currently intends to play and (2) further reducing the set E(t) based on proposals made by its partner (which gives it the potential to more quickly find cooperative solutions with cooperative partners). Full details about S++ and S# as they were implemented for these studies are given by Crandall et al. (2018).

**Figure 3:**
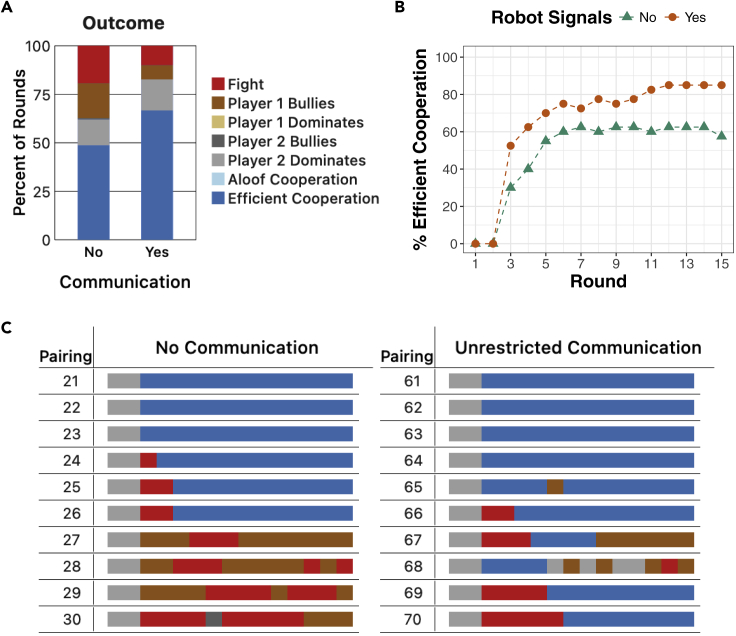
Results of bot-bot pairings in the Block Dilemma (A) The percentage of rounds across all pairings that resulted in each category of round outcome. Results are averaged over 40 pairings in each condition. (B) The percentage of pairings resulting in *Efficient Cooperation* in each round. (C) The outcome of all 15 rounds of 10 (out of 40) representative pairings in each condition. For example, in Pairing #24, the second bot dominated the first bot for the first two rounds; the bots then fought in round 3, but then converged to *Efficient Cooperation* for the remaining 12 rounds.

Figure 3 shows the behavior of two agents (or bots), both of which followed S# to select blocks, when paired together in the Block Dilemma. Results are shown for both when communication was and was not permitted. Like pairs of people, these pairs of bots often quickly learned to cooperate with each other in this game. Additionally, like humans, the bots tended to cooperate more often with communication than without communication, which led to higher payoffs.

However, when compared with the human-human results shown in Figures 2, Figure 3 also illustrates several differences between the behavior of bots and humans in the Block Dilemma. First, whereas pairs of humans typically failed to achieve *Efficient Cooperation* without communication, these bots often converged to *Efficient Cooperation* (Figures 3A and 3C). In fact, *Aloof Cooperation* among these pairs of bots was essentially non-existent with and without communication, whereas *Efficient Cooperation* was non-existent between humans without communication, although it was somewhat common with communication. Second, bullying (unfair) outcomes, in which one player continually gets the set of squares, was more common among these bots than among people. This seems to be particularly true when communication is not permitted. Third, the bots were somewhat slower to converge than humans (Figure 3B). As such, they received lower payoffs on average in early rounds of the interaction, although they quickly recover after that.

These results indicate that human behavior and machine behavior appear to be somewhat aligned when communication between players is unrestricted. However, in the absence of communication, human and machine behaviors appear to be misaligned. Pairs of people avoided risk and unfairness in the absence of communication at the expense of efficiency (i.e., receiving higher payoffs), whereas pairs of bots initially prioritized efficiency due to S#’s strategy selection mechanism (see Equation 1) rather than risk and fairness regardless of whether communication was permitted or not.

These results indicate that, in a very real sense, the machine's behavior used in this study was superior in the Block Dilemma than human behavior when communication was not possible, as pairs of machines often converged to more profitable solutions than pairs of people. On the other hand, the misalignment between machine and human behavior could have potential ramifications on the ability of humans and machines to cooperate with each other. Can S# convince people to efficiently cooperate with it given this misalignment? We explore this question in the next section.

### Study 3: human-robot behavior in the Block Dilemma

To observe human-robot cooperation in the Block Dilemma, we conducted another user study in which a Sawyer robot, which we call Tibor, interacted with people in a 15-round Block Dilemma (Figures S1C and Video S1). Tibor was enhanced with additional cameras and a microphone so it could better perceive its environment and communicate its strategy and internal state to people when playing the Block Dilemma. The robot was equipped with a screen face, through which it could express emotions, and a pneumatic gripper, which allowed it to grasp and move blocks. It was also equipped with safety features that allowed it to operate in close proximity to people. Additional information about Tibor's hardware and the software system it used to play the Block Dilemma in this study are provided in the Supplemental information (see Transparent methods: SM 3).

**Figure 4:**
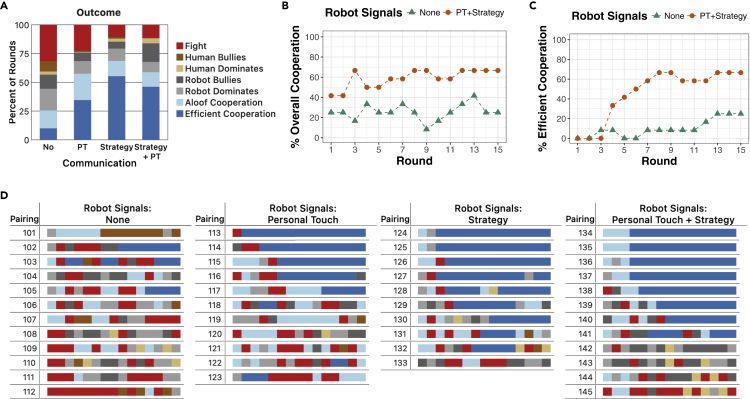
Results of human-robot pairings in the Block Dilemma (A) The average percentage of rounds that resulted in each category of round outcome. (B) The percentage of interactions ending in either *Efficient* or *Aloof Cooperation* in each round. (C) The percentage of interactions ending in *Efficient Cooperation* in each round. (For simplicity, only the no signals and Strategy + Personal Touch conditions are shown in B and C). (D) The outcome of each of the 15 rounds of each pairing.

Video S1. Illustration of Tibor playing the Block Dilemma with a human partner, related to Study 3 and Figure 4

In addition to using S# to determine which blocks to select, Tibor was equipped with custom features to communicate with people in the Block Dilemma. First, Tibor verbalized its strategy, its expectations of people, and its feelings (a.k.a., reflections on its internal state) via a speaker. These verbal signals were designed consistently with the speech system produced by S# in previously published studies (Oudah et al., 2015; Crandall et al., 2018). Second, Tibor verbalized statements related to game flow, such as requesting the human's help recovering from known failures. Third, Tibor expressed its emotions through facial expressions, displayed on its face (Figure S2). Tibor's facial expressions included joy, surprise, anger, sadness, or no feeling (neutral). The facial expression displayed on Tibor's face was selected to be consistent with the internal state of S#. Fourth, in an attempt to make Tibor appear conscious of its environment, we animated Tibor's mouth and eyes so that it blinked and tracked people's faces and hand movements. Additional details about Tibor's verbal and non-verbal communication signals are provided in the Supplemental information (see Transparent methods: SM 3).

Tibor's human partner in the game could likewise communicate verbally with Tibor by talking into a microphone. To aid Tibor's understanding of its human partners, human speech was limited to the set of phrases listed in Table S3. This set of speech acts allowed human participants to propose solutions (e.g., “This round, I get the squares”) and express satisfaction or dissatisfaction with proposals and outcomes (e.g., “That's not fair.”). Before each round, Tibor processed all the proposals made by the human during the previous round and used these as input to S#.

As in the human-human and machine-machine studies reported in the previous sections, we studied human-robot cooperation in the Block Dilemma under different communication conditions. In this case, the communication conditions were defined by the communication signals conveyed by Tibor, which we divided into two categories: (1) signals dealing with strategy, including both proposed solutions and its expectations of its partner's behavior, and (2) signals designed to communicate emotion and the robot's awareness of the environment. We refer to the first set of signals as *Strategy Signals*, and the second set of signals as *Signals of Personal Touch*. Additional details for how Tibor's signals were categorized in this study are given in the Supplemental information (see Transparent methods: SM 3, and Tables S1 and S2).

This user study followed a 2×2 between-subjects design in which strategic communication (off or on) and personal touch (off or on) were the independent variables. In conditions under which strategic communication was enabled, Tibor explicitly expressed Strategy Signals (as they were selected by S#), whereas when they were disabled, Tibor did not express those signals. Likewise, in conditions under which personal touch was disabled, Tibor's face was static (neutral expression with no blinking or mouth movements), it would just look straight forward. Likewise, it did not voice speech acts related to feelings or its awareness of the environment (except speech required to recover from errors to keep the game flowing). Tibor's communication varied based on the condition of the study, whereas the human player was able to voice speech to Tibor in all conditions, which can impact Tibor's behavior (because S#, the strategic algorithm used by Tibor, uses proposals from its partner to adapt its strategy).

Forty-five people participated in the study. Each participant was randomly assigned to one of the four conditions: *No communication*, *Personal Touch*, *Strategy*, or *Strategy + Personal Touch*. The same protocol was followed for each participant as in the human-human study described in Study 1, except that only one person participated in the study at a time. The participant in this study was always assigned to be player 1 (the player who selected the block first in each round), and Tibor was player 2. Additional details about the study are given in the Supplemental information (see Transparent methods: SM 2–4 and Figure S3).

In the studies of human behavior and machine behavior discussed in the previous sections, we observed that both people and S# often converge to *Efficient Cooperation* in the Block Dilemma when communication is permitted. As such, we would expect a high degree of human-robot cooperation given unrestricted communication. However, when communication was not permitted, pairs of people typically converged to *Aloof Cooperation* (and never to *Efficient Cooperation*) in the Block Dilemma, whereas S# in self-play often converged to *Efficient Cooperation* (and never to *Aloof Cooperation*). Thus, there is more uncertainty regarding what will happen when people and Tibor are paired together in the Block Dilemma with restricted communication. In such circumstances, will the human or robot adapt to the other, or will fighting or bullying punctuate the relationship?

The results of our study in which people interacted with Tibor in the Block Dilemma are summarized in Figure 4. These results show that when all robot signals (i.e., both Strategy Signals and Signals of Personal Touch) were disabled, people cooperated with Tibor in only about 25% of the rounds (Figures 4A–4C). In fact, without robot communication, only three of twelve participants established *Efficient Cooperation* with Tibor for at least two consecutive rounds (Figure 4D). In all other conditions, cooperation rates were more than double, including relatively high levels of *Efficient Cooperation* when Strategy Signals were enabled. A Mann-Whitney test indicates that the robot's Strategy Signals had a significant impact on human-robot cooperation (for overall cooperation: U=369, p=0.009; for *Efficient Cooperation*: U=380, p=0.003). As a result, the average per-round payoff of both the human ($0.76 versus $0.46) and the robot ($1.04 versus $0.76) was higher when the robot expressed Strategy Signals than when it did not. Signals of Personal Touch alone likewise raised cooperation over no signals at all (significant for overall cooperation: U=19, p=0.007; marginally significant for *Efficient Cooperation*: U=33.5, p=0.061).

In short, whereas S# in self-play often reached *Efficient Cooperation* in the Block Dilemma without explicitly communicating, it typically was unable to elicit *Efficient Cooperation* from human players without explicitly communicating with them. Similarly, whereas pairs of human players often achieved *Aloof Cooperation* without communicating with each other, human-robot pairs did not achieve high levels of *Aloof Cooperation* when the robot did not communicate. The behavioral difference between the players (wherein pairs of machines tend to find the risky but efficient solution, whereas pairs of people tend to find the less risky but less efficient solution) appears to create a disconnect that produces a dysfunctional relationship wherein both players lose money in many rounds (Fight).

The phrases chosen by participants to communicate with the robot are helpful in understanding human behavior in the Block Dilemma. Across the four conditions, 36 of 45 participants proposed (or agreed to) *Efficient Cooperation* (17 of 23 in conditions without Strategy Signals, 19 of 22 in conditions with Strategy Signals) at least once during their interaction with the robot. However, 39 of the 45 participants also voiced other forms of proposals. These other proposals were most often some form of *Aloof Cooperation*, although proposals to “always get the squares” or to let the robot “always get the squares” were also made. That said, *Efficient Cooperation* was only the first proposal made by just 12 of 45 participants—most participants voiced some other proposal first. Together, these results indicate that participants typically (but not always) identified *Efficient Cooperation* as a possibility. However, they either did not always think it was ideal or they did not believe they could get the robot to carry it out.

The robot's communication signals (to its human partner) appear to have been influential in turning human proposals of *Efficient Cooperation* into actual *Efficient Cooperation*. Of the 28 pairings in which the human participant discussed *Efficient Cooperation* with the robot when Strategy Signals or Signals of Personal Touch were given by the robot, 23 pairings eventually arrived at *Efficient Cooperation*. On the other hand, in the condition with no robot signals, only three of the eight pairings (Pairings #102, #103, and #105) in which humans proposed *Efficient Cooperation* to the robot eventually reached *Efficient Cooperation*. The other five participants failed to establish *Efficient Cooperation* with the robot for various reasons. The human participant in Pairing #104 proposed *Efficient Cooperation* in round 6. The human participant tried to carry out the proposal (seeking to get all the squares in round 6 and all the triangles in rounds 7 and 8), but the robot did not conform (but instead tried to bully the human). On the other hand, the human participants in Pairings #110 and #112 proposed *Efficient Cooperation*, but then refused to give the robot the squares when it was the robot's turn to get them. This led to frequent fighting. The human participant in Pairing #109, on the other hand, simply went for a mixed set after proposing *Efficient Cooperation* when the robot intended to allow the human to get all the squares. The eighth participant (in Pairing #106) did not propose *Efficient Cooperation* until round 15, so it is unclear whether or not *Efficient Cooperation* would have emerged had the interaction lasted longer. Thus, the lack of robot communication appears to have made it difficult for the players to negotiate and coordinate the risky and somewhat sophisticated solution of *Efficient Cooperation* in the Block Dilemma.

Communication logs also indicate differences in how the robot and human viewed fairness. In the 22 interactions in which the robot's Strategy Signals were enabled, the robot proposed that it should always get all the squares in 12 of them (doing so a total of 32 times in those interactions). On the other hand, the human participant explicitly proposed that they should always get all the squares in just four of those interactions (all 22 participants made at least one proposal in these conditions). These differences further illustrate the differences in the robot's and the humans' overall strategies in relationship to fairness. Interestingly, these differences appear to have contributed to an imbalance in earnings, with the robot earning $0.90 per round (over all conditions) and human participants earning just $0.61 per round.

These results suggest that, rather than focusing exclusively on the efficiency of solutions, machines must also consider other attributes of solutions (such as risk and fairness) to establish cooperative relationships with people. Machines can do this by either aligning their behavior with people (even if it means playing less efficient solutions) or finding ways to making these efficient solutions appear more appealing and achievable to people.

### Study 4: human-disguised AI behavior in the Block Dilemma

Results of our human-human study (Study 1) showed that humans tended to converge to *Aloof Cooperation* when they could not communicate. On the other hand, in the user study presented in the last section, human-robot cooperation was substantially lower, punctuated with higher rates of dysfunction, when the robot did not communicate. However, the cause of these differences is unclear as the conditions of these studies differed in three ways. First, the identity of the playing partner (human or robot) was visible to the players in both studies, and this has been shown to cause human bias that results in lower cooperation when people interact with artificial agents than with people (Ishowo-Oloko et al., 2019). Second, participants were allowed to speak to their partner (the robot) in the human-robot study, whereas they were not allowed to speak to each other in the human-human study when communication was not permitted. Third, the strategy used by humans differed from that of S#. Hence, we cannot determine whether the physical identity of one's partner (human or robot), the communication condition, or the partner's strategy (or all three) caused the primary differences in these outcomes.

To address which of these factors is primarily responsible for the differences, we conducted a final user study. This study was identical to the no-communication condition of the human-human study reported in Study 1 (see the configuration shown in Figure S1B), except that the second human (player 2) was a confederate that simply selected blocks as dictated by S#. The 10 study participants involved in this study were unaware that their partner was following actions suggested by an algorithm. Thus, this study differed from the no-communication condition of the human-human study only with respect to the strategy used by player 2. We refer to this condition as the *Disguised-AI* condition.

**Figure 5:**
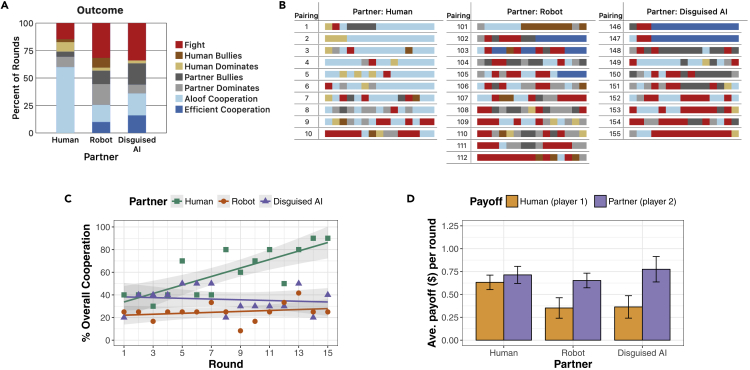
Comparison of outcomes with non-communicating partners (A) The average percentage of rounds over all participants that resulted in each category of round outcome when people were paired with other people, Tibor, and a disguised AI, each of whom did not communicate. (B) Summaries of each round for all pairings in each condition. (C) The percentage of interactions ending in either *Efficient* or *Aloof Cooperation* in each round. For better visualization, fitted lines show a linear model of the data, with shaded areas representing 95% confidence intervals of the model's fit. (D) The average payoffs received by people and their partners. Error bars show the standard error of the mean.

The results of this study, alongside the results from the no-communication and no-robot-signals conditions of the human-human and the human-robot studies, are shown in Figure 5. A visual inspection of the average round outcome (Figure 5A), the distribution of outcomes in individual interactions (Figure 5B), and overall cooperation rates over time (Figure 5C) show substantial similarity between human-robot (no signals) and human-disguised AI pairings, and substantial differences between those studies and the human-human results. Mann-Whitney tests comparing human-human and human-disguised AI pairs show a marginally statistically significant difference between rates of overall cooperation (U=27, p=0.087), and a statistically significant difference between rates of Fighting (U=82.5, p=0.014). As a result, the human's payoffs were only about half as much when associating with a robot or disguised AI than when associating with another person (Figure 5D). On the other hand, due to high rates of the robot dominating and bullying its human partners, the human partner's payoffs varied little.

Thus, although these results do not rule out the possibility that the differences in the communication condition or the knowledge of the robot as a partner (rather than a human) contributed to differences in cooperation rates, they suggest that the differences we observed in the human-human and human-robot are primarily driven by the strategy used by the robot (and disguised AI). The robot's inability to establish cooperative relationships with people without communication is due to its failure to align its own behavior with human behavior (or vice versa).

## Discussion

In this article, we analyzed and studied various enablers and barriers to human-robot cooperation. Prior work has shown that the algorithm S# establishes and maintains cooperative relationships with people in a variety of repeated games when it is allowed to verbally communicate with people (Oudah et al., 2015, Oudah et al., 2018; Crandall et al., 2018). Although the ability of machines to cooperate with people at rates that mirror human cooperation is encouraging, these same studies illustrate that S# often fails to establish cooperative relationships with people when it cannot verbally communicate with them. In fact, S#’s ability to cooperate with like-minded machines without verbal communication is substantially higher than its ability to cooperate with people (Crandall et al., 2018).

To better understand how to design machines that elicit cooperation with people in repeated games (even without verbal communication), we conducted a series of simulations and user studies in the Block Dilemma, a rich resource-sharing scenario that has many solutions that vary with respect to efficiency, fairness, and risk. In particular, the game contains a set of inefficient, fair, and less risky solutions (which we refer to as *Aloof Cooperation*); an efficient, fair, and more risky solution (which we refer to as *Efficient Cooperation*); and a set of efficient but less fair solutions that provides incentives for players to seek their own welfare over that of their partner.

Although both strategic concerns (i.e., machine behavior (Rahwan et al., 2019)) and human psychological biases can impact human-robot cooperation, our results indicate that strategic concerns have the most impact on human-robot cooperation in the Block Dilemma. A summary of the outcomes of these studies is shown in Table 1. When players are allowed to communicate with each other, human behavior and machine behavior (as defined by S#) are aligned—both pairs of humans and pairs of machines tend to converge to *Efficient Cooperation*. Unsurprisingly given this finding, human-machine pairs also frequently converge to *Efficient Cooperation* when they can communicate. On the other hand, without communication, human behavior and machine behavior are not aligned, as pairs of humans tend to converge to *Aloof Cooperation,* whereas pairs of machines converge to *Efficient Cooperation*. As a result, when people and these machines are paired together in the Block Dilemma, they often fail to cooperate when communication is restricted due to their strategic differences.

**Table 1:** Predominant behavioral tendencies observed in the Block Dilemma

	Human Dyads	Machine Dyads	Human-Machine Dyads
With communication	*Efficient Cooperation*	*Efficient Cooperation*	*Efficient Cooperation*
Without communication	*Aloof Cooperation*	*Efficient Cooperation*	*Fight*

In short, human behavior in the Block Dilemma, which is based on perceptions of risk, fairness, and efficiency, stands in contrast to that of S#, whose sole focus is on the efficiency of solutions. As *Aloof Cooperation* in the Block Dilemma does not produce payoffs that initially meet its aspiration level, S# does not consider playing it initially. Thus, when people's assessments of risk and fairness discourage them from considering more efficient solutions, S# is unable to consistently find common ground with people until its aspiration level drops substantially (at which point it does consider less efficient solutions).

Analyses of communication patterns in these studies indicate that although communication is less important to establishing machine-machine cooperation, human players rely on communication to find and play efficient but risky outcomes. In scenarios in which fair and efficient solutions have high risk, communication helps human players to (1) discover efficient solutions, (2) determine that the other player is also aware of these solutions, and then (3) negotiate which solution to play and coordinate how to play it. Without such communication, efficient cooperation can seem to be too risky to human players, even though committing to such solutions could yield higher payoffs (payoffs that are often achieved by pairs of machines without communication).

Other learning algorithms differ from S#, whereas machine behavior is typically produced using the concept of maximizing expected utility (Russell and Norvig, 2009) (which is closely related to the efficiency of payoffs). Because people often do not maximize expected utility (e.g., prospect theory shows that people weigh risks differently than benefits, Kahneman and Tversky, 1979), other algorithms designed with respect to this methodology will also likely encounter many of the same issues as S#. The results of this article suggest the need for a different approach, one that carefully balances the efficiency, fairness, and risk of solutions in a way that better allows the machine to relate with people, and vice versa.

A first potential approach is based on computing detailed models of human behavior (e.g., Peled et al., 2015) and then computing optimal solutions with respect to those models. This approach falls under the umbrella of opponent modeling (Albrecht and Stone, 2018). When such models are accurate, opponent modeling can produce algorithms that conform with human behavior, such as playing less efficient (less risky) solutions in the Block Dilemma when communication is restricted. However, such approaches risk robbing the machine of opportunities to achieve more efficient solutions (Crandall et al., 2018). Furthermore, obtaining accurate models of people for arbitrary scenarios (rather than situation-specific models built by data gathered in specific scenarios) remains extremely difficult despite a myriad of approaches that have been investigated over many years (Albrecht and Stone, 2018; Crandall, 2020).

A second (alternate) approach to creating machines capable of aligning machine and human behavior embraces the advantages of machines in computing efficient solutions. Such machines would still advocate for more efficient solutions, but would do so in a way that would help people be more prone to considering these more efficient (albeit potentially risky) solutions when warranted. By better considering and balancing efficiency, fairness, and risk in machine behavior in this way, we believe that machines can overcome important barriers to human-machine cooperation.

### Limitations of the study

The insights gleaned from these studies were obtained for a single scenario (the Block Dilemma). Furthermore, the human-robot interactions were considered in the context of a single robot system. Similar kinds of studies that consider different scenarios (with potentially different payoff structures and characteristics of interaction) and robot systems may produce different kinds of outcomes. Of particular mention is the strategy used by the robot to make decisions and communicate with people. In this article, our robot used the algorithm S# (Crandall et al., 2018) to make decisions and to communicate with people. However, other algorithms with alternative strategic characteristics are likely to induce different outcomes (including different forms of cooperation) when interacting with people (e.g., Oudah et al. (2015)). Our studies highlight the need for further studies that analyze how the strategic nature of the algorithm used by the robot impact human-robot cooperation.

### Resource availability

#### Lead contact

Further information and requests for resources should be directed to and will be fulfilled by the Lead Contact, Jacob Crandall (crandall@cs.byu.edu).

#### Materials availability

The study did not generate new unique materials.

#### Data and code availability

The data and code associated with the studies in this manuscript will be supplied upon request by the lead author. Please send email to crandall@cs.byu.edu.

## Methods

All methods can be found in the accompanying Transparent Methods supplemental file.

## Supplemental information

Document S1. Transparent methods, Figures S1–S3, and Tables S1–S3

## Footnotes

Supplemental Information can be found online at https://doi.org/10.1016/j.isci.2020.101963.
