# Supplementary material for: Confronting barriers to human-robot cooperation: balancing efficiency and risk in machine behavior
Source: iScience. 2020 Dec 17;24(1):101963. doi: 10.1016/j.isci.2020.101963 (PMC7797565; doi:10.1016/j.isci.2020.101963)
Supplement: Document S1. Transparent methods, Figures S1–S3, and Tables S1–S3 [file mmc1.pdf]

iScience, Volume *24*

Supplemental Information

Confronting barriers to human-robot cooperation: balancing efficiency and risk in machine behavior

Tim Whiting, Alvika Gautam, Jacob Tye, Michael Simmons, Jordan Henstrom, Mayada Oudah, and Jacob W. Crandall

Supplementary Information

1. Supplementary Figures and Tables
2. Transparent Methods
3. Supplementary References

Supplementary Figures and Tables

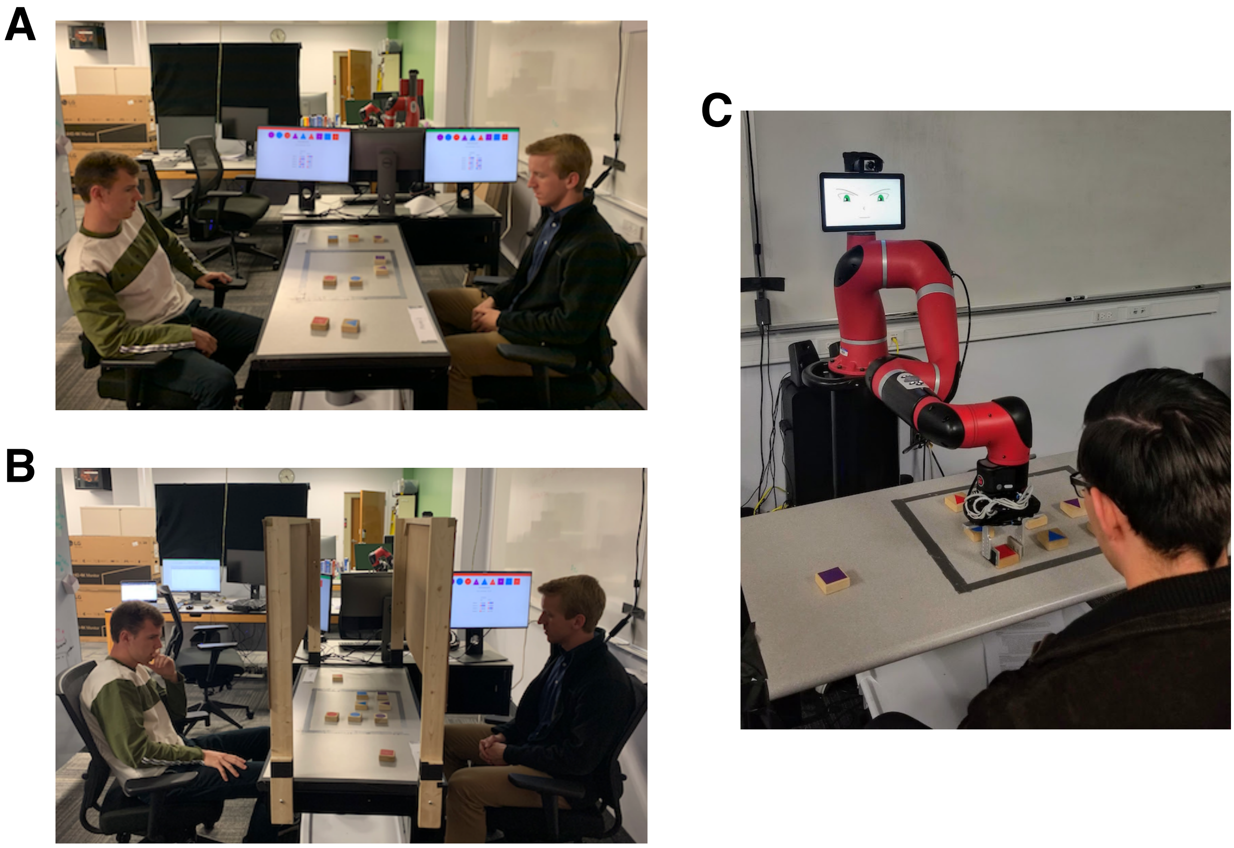

Figure S1: Physical setups for the various user studies conducted in this research. Related to Studies 1, 3, and 4 and Figures 2, 4, and 5. (A) The physical setup for the *unrestricted-communication* condition of Study 1. (B) The physical setup for the *no-communication* condition of Study 1 and the human-disguised AI condition of Study 4. In these conditions, physical barriers were placed on the table to keep the study participants from seeing each other's faces. (C) The physical setup used in Study 3, in which study participants played the Block Dilemma with Tibor (a Sawyer robot).

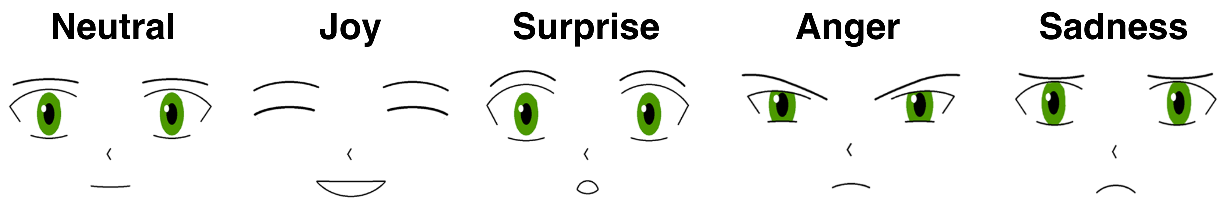

Figure S2: The five facial expressions used by Tibor. Related to Study 3 and Figure 4.

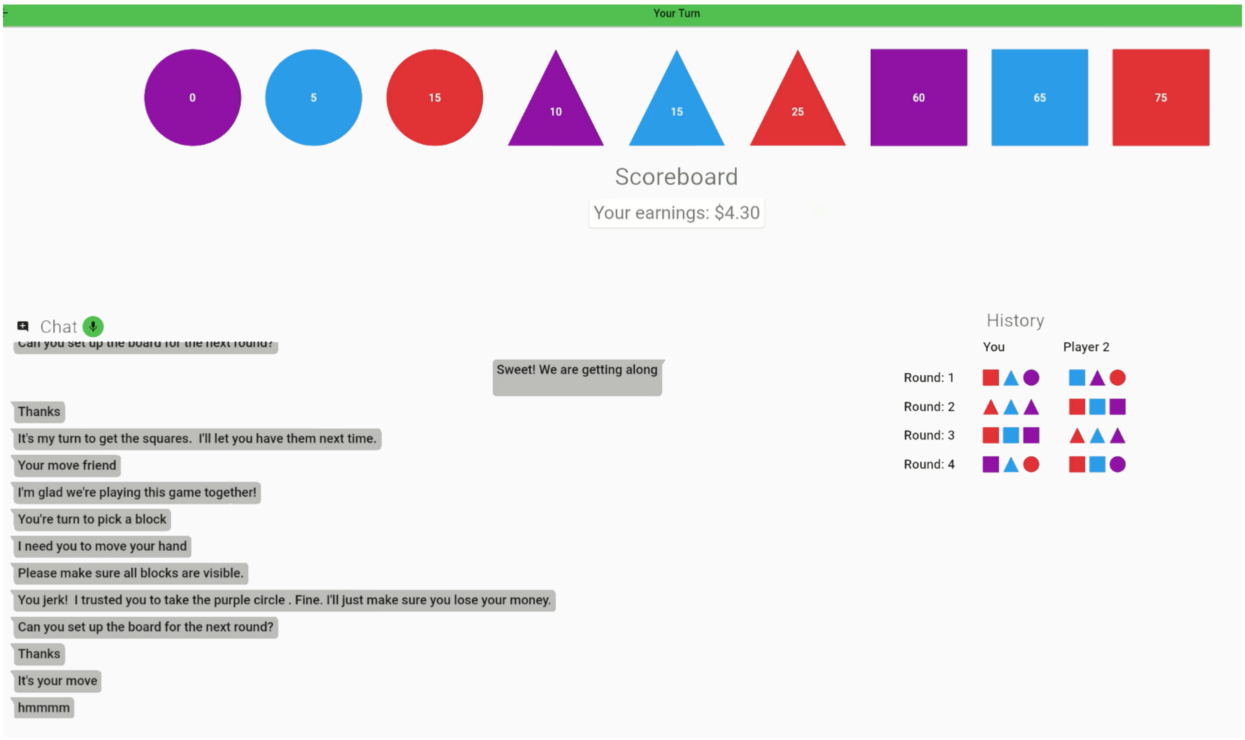

Figure S3: The scoreboard interface displayed to participants to help them keep track of their interaction. Related to Studies 3 and 4 and Figures 4 and 5.

Table S1: Example speech acts used by Tibor to communicate its strategy and to establish personal touch. Related to Study 3 and Figure 4.

**Scenario**	**Strategy**	**Personal Touch**
Between rounds	- I always get all of the squares.<br>- Let's take turns getting the better blocks.	- I'm enjoying this.<br>- Things are not going my way.
Cooperation has emerged	- Let's continue this.<br>- That's what I wanted.	- Excellent.<br>- Great!
Partner deviated	- I'll just make sure you lose your money.<br>- I'm going to make you regret doing that.	- You jerk! Be nice to me.<br>- Serves you right, jerk.

Table S2: Example speech acts used by Tibor to recover from errors. Related to Study 3 and Figure 4.

**Scenario**	**Personal Touch**	**Recovery (all conditions)**
Grasping error		- Please pick up the red square block for me<br>- Oops. Let me try again.
Vision error	- I hate not seeing perfectly.<br>- Vision is such a good thing to have working.	- Please make sure all blocks are visible.<br>- I have to reset my vision subsystem.<br>- Your hand is in the way of my vision.

Table S3: The set of speech acts provided to human players to communicate with Tibor. Related to Study 3 and Figure 4.

ID	Message
1	I want the [squares/triangles/circles]
2	You can have the [squares/triangles/circles]
3	I always get the [squares/triangles/circles]
4	I want the [red/blue/purple] blocks
5	You can have the [red/blue/purple] blocks
6	I always get the [red/blue/purple] blocks
7	Let's each get a mixed set
8	Let's always get mixed sets
9	You can always have the [squares/triangles/circles]
10	You can always have the [red/blue/purple] blocks
11	Let's take turns getting the [squares/triangles/circles] and [squares/triangles/circles]
12	Let's take turns getting the [red/blue/purple] and [red/blue/purple] blocks
13	Yes, I accept your last proposal
14	No, I don't accept your proposal
15	Sweet! We are getting along
16	You will pay for this
17	Curse you
18	In your face
19	I forgive you
20	Give me another chance
21	Excellent
22	We can both do better than this
23	That's not fair
24	Do it or I will punish you
25	You misunderstood me
26	You betrayed me
27	I don't trust you

Transparent Methods

SM 1: The Block Dilemma

The Block Dilemma is a resource-sharing scenario in which two players repeatedly interact with each other. In this section, we describe the dynamics of the game and its strategic characteristics. In subsequent sections, we describe a series of simulations and user studies we conducted with the Block Dilemma to study human-machine cooperation.

Game Description

In the Block Dilemma, two players interact in a series of rounds. In each round, the players play an extensive-form game in which they take turns selecting blocks from the set of nine blocks shown in Figure 1A (in the main paper), with player 1 always selecting a block first in each round. The round ends when each player has selected three blocks. The number on each block indicates its monetary value in cents (USD). When a player's three blocks form a valid set (i.e., all their blocks have the same color, or have the same shape, or have nothing in common), then the player's earnings in the round are the sum of the numbers on their blocks. The round earnings of a player that fails to collect a valid set of blocks is the sum of the numbers on their blocks divided by −4, meaning that a player that does not get a valid set loses money in that round.

Payoff Space

To illustrate how sets of blocks equate with payoffs (money earned) in the Block Dilemma, several common round outcomes are shown in Figure 1C (main paper). However, many other round outcomes are possible (673 to be exact); the full set of round outcomes are plotted in Figure 1B. We categorize these round outcomes into six different categories. Two categories consist of outcomes in which one of the players gets all of the squares (labeled *Player 1 Bullies* and *Player 2 Bullies*, respectively). The set of squares is the most profitable valid set, and is worth $2.00. However, this leaves the other player with a relatively poor set of blocks worth at best $0.50 (when the other player selects the triangles; see example #1 in Figure 1C).

Because outcomes in which one player gets all the squares are seemingly unfair, attempts to get all of the squares may be met with opposition from the other player. Example #2 in Figure 1C illustrates a scenario in which player 1 selects the red square first and then player 2 selects the red triangle. Player 1 then reveals its intention to try to get all of the square by taking the blue square. Player 2 prohibits this by selecting the purple square. Player 1 then retaliates and takes the blue circle, which is the block that player 2 needed to complete a valid set. As a result, neither player gets a valid set, resulting in both players losing money in the round. We label this outcome a *Fight*, noting that 452 of the 673 possible round outcomes are likewise *Fights* in which neither player gets a valid set (hence each loses money in the round).

To avoid fights, players may seek to get other valid block sets, such as the *mixed sets* (in which no block has the same shape or color) shown in Example #3 in Figure 1C. Each mixed set is worth $0.90, and it is possible for both players to get such a set in a round. Other pairs of block sets are also possible, such as when one player gets all of the red blocks (worth $1.15) while the other player gets all of the blue blocks (worth $0.85). We categorize outcomes in which both players get valid sets but neither player gets all of the squares as *Aloof Cooperation*, since these outcomes require the players to coordinate their selections, but do not require the players to engage substantially beyond that. The players just need to coordinate which sets they pursue so that they do not need the same block to complete their sets (see Example #4 in Figure 1C for a scenario in which the players fail to coordinate).

Outcomes labeled as *Aloof Cooperation* are more fair than the bully outcomes, in that the differences between the players' earning in a round are lower. However, as illustrated in Figure 1B, none of the outcomes labeled as *Aloof Cooperation* produce payoffs on the Pareto boundary of the convex polygon that defines the game's convex hull (the grey-shaded region in the Figure). Only round outcomes in which one player gets all of the squares reside on this Pareto boundary, and these outcomes are not fair. However, a fair and Pareto efficient average joint payoff is produced over a series of rounds when players take turns getting the squares and triangles (Example #5 in Figure 1C). This joint behavior, which we call *Efficient Cooperation*, yields a player $2.00 in one round followed by $0.50 in the next round, resulting in an average per-round payoff of $1.25. As such, *Efficient Cooperation* is more profitable for both players than any form of *Aloof Cooperation*.

Despite being better for both players than *Aloof Cooperation*, seeking the compromise of *Efficient Cooperation* is risky for a variety of reasons. First, this solution exposes the players to risk since it requires one player to receive a low payoff in one round with the hope that the other player will allow them to receive a high payoff in the next round. Thus, opening one's self to the opportunity of *Efficient Cooperation* exposes a player to being exploited. Second, seeking *Efficient Cooperation* when the other player will not agree with it can be costly. For example, by selecting two squares, a player exposes itself to the potentiality of a high loss if the other player decides to take the third square (see Example #2 in Figure 1C). The other player may interpret the attempt to get all of the squares in a round as greedy (it is easily conflated with a desire to *bully*), which can lead to conflict and *Fights*. In short, seeking *Efficient Cooperation* is risky, similar to *hunting stag* instead of *hunting hare* in a Stag Hunt (Skyrms., 2004).

Game Theoretic Analysis

Since neither player can get a valid set in a round without the cooperation of the other player, both players' minimax values are below 0.0. If both players use minimax (Nash, 1928) to select blocks, player 1 would select all of the purple blocks while player 2 would select all of the blue blocks (under the assumption of a reasonable tie-breaking strategy), resulting in round payoffs of \$0.70 and \$0.85, respectively. This solution is a rather inefficient instance of *Aloof Cooperation*. We did not observe any instance of this outcome in any of the studies reported in this paper.

The sub-game perfect Nash equilibria (Nash, 1950; Gintis, 2000) of a single round of the Block Dilemma correspond to the cases in which each player gets a mixed set of blocks, resulting in round earnings of \$0.90 to each player. Other Nash equilibria of the round of a game do exist, albeit they are not sub-game perfect. If one player can convince the other player that they are not fully rational, they can bully the other player so that they get a higher payoff. For example, player 1 could threaten player 2 that if player 2 does not let them get all of the squares, then they will not let player 2 get a valid set. If player 2 believes this threat, they would be better off selecting only triangles (thus letting player 1 take all of the squares). However, such a threat is risky. Selecting multiple squares could result in a large loss if player 2 does not conform. The threat is also irrational, as carrying it out would not be in player 1's self interest with respect to the current round's payoffs. As such, this equilibrium strategy is not sub-game perfect.

When the game is played repeatedly with a reasonably high probability of the players interacting again after each round, the folk theorem (Gintis, 2000) shows that the game has many Nash equilibria. These Nash equilibria all produce payoffs within the game's convex hull (the grey-shaded region in Figure 1B) that give both players higher payoffs than their maximin values. However, not all points in this space are equally valuable, nor will self-interested players agree on which outcome in this space is most desirable. One possible point of compromise that is both fair and Pareto optimal is the Nash Bargaining Solution (Nash, 1950), which corresponds to *Efficient Cooperation* wherein the players take turns getting the squares and triangles.

SM 2: User Study Details

Procedures

For each participant, the study[1] proceeded as follows:

1. The subjects participated in the study in groups of two. Each pair was assigned one of the two study conditions based on a previously determined schedule defining the order in which pairs were to be assigned to conditions. Figure S1A-B shows the setup for human-human pairs in both conditions, while Figure S1C illustrates the physical setup for human-robot pairings.
2. The participants were separately instructed on how to play the Block Dilemma and how they were to be paid. The participants were allowed to ask whatever questions they desired. At the end of this training, each participant was asked questions verifying their understanding of the basic rules of the game. Any questions missed by the participants were reviewed and misconceptions were clarified.

[1]The user studies in this paper were approved by Brigham Young University's Institutional Review Board (IRB).

3. Each participant played a single 15-round game with their assigned partner (participants were involved in only a single study each). Participants were not told how many rounds of the game they would play. In each round, player 1 selected a block first. Throughout the game, the history of moves was displayed on a screen visible to each participant. The screen also showed the participant the current running tally of the money they had earned over all rounds played so far. The game typically lasted between 20-30 minutes.
4. Upon completing the 15-round game, the participant completed a post-game survey questionnaire, consisting of questions (answered on a 5-point Likert scale) in which participants rated themselves and their partner with respect to seven attributes: trustworthiness, vengeance, predictability, cooperativeness, deviousness, propensity to bully, and selfishness.
5. The participants were paid through a money voucher, which they could redeem immediately for cash. The amount of money paid to the participants was equal to the amount of money earned in the game, except that each participant was given $10 (USD) at a minimum.

Demographic Information of Study Participants

Human-human study (Study 1): Forty students (31 males, 9 females) were recruited from the Brigham Young University campus in Provo, UT, USA to participate in this study. The average age of the participants was 22.8 years. Eight-five percent of the study participants were majoring in a degree related to science or technology and 93% had some degree of familiarity with strategic games. In order to avoid biases, pairs for each game were formed such that the respective players were not familiar with each other prior to the study.

Human-robot study (Study 3): Forty-five students (32 males, 13 females) were recruited from the Brigham Young University campus in Provo, UT, USA to participate in this study. To avoid learning effects, these participants were distinct from those participating in the other studies. The average age of the participants was 22.7 years. Sixty-six percent of the study participants had a science/technology background and 98% had some degree of familiarity with strategic games. Several additional people also participated in the study. However, due to system errors and failures (which either prohibited the 15 rounds from completing or otherwise compromised the results), we discarded the results from those interactions.

Human-Disguised AI study (Study 4): Ten participants (6 males, 4 females) were recruited from the Brigham Young University campus in Provo, UT, USA to participate in this study. To avoid learning effects, these participants were distinct from those participating in the other studies. The average age of the participants was 22.2 years. Eight of the ten participants came from STEM backgrounds.

SM 3: Robot System

Tibor is a Sawyer robot developed by Rethink Robotics which we enhanced with additional cameras and a microphone so it could better perceive its environment and communicate strategy and personal touch. In this section, we provide additional information about Tibor's behavior and processes.

Vision and Grasping

In our studies, Tibor perceived its environment using a wide-angle camera for face tracking and a Kinect2 Wiedemeyer (2015) for detecting and localizing blocks and recognizing hand movements over the table. The wide-angle camera, mounted to Tibor's head, used a Haar cascade in combination with a Kalman filter to detect and track faces. The Kinect2 was mounted from the ceiling, giving a bird's-eye view of the table, player and blocks. Hands and blocks were detected using a combination of color and shape detection using OpenCV Bradski (2000), and then localized by indexing into a point cloud which yielded the respective (x, y, z) coordinates of the blocks or hands. The block coordinates were then passed into the manipulation system, which uses MoveIt Sucan and Chitta (2013) for motion planning. Hand detection was reported to the agent so it could decide when it was safe to engage in a manipulation task.

Error Detection and Recovery

The robot sometimes failed to identify or pick up a block because both vision and grasping were subject to substantial hardware, software, and environmental complexities. Thus, we created various recovery mechanisms that would allow the robot to keep its interaction going in the presence of errors. For example, when failing to pick up a block the robot reattempted the action once. If still unsuccessful, Tibor would then ask the human for assistance instead.

In event of an unexpected failure of the vision subsystem, the system would automatically restart it. It was also able to detect known reasons for failure and propose potential recovery mechanisms. For example, Tibor would ask the player to move their hand when it sensed that the blocks were occluded by the player's hand.

Tibor's Communication

Tibor used both verbal communication (speech acts) and non-verbal communication (gaze and facial expression) capabilities. This section describes them one by one, and then discusses more in depth how the various signals were categorized as either *Personal Touch* or *Strategy* signals.

Speech Acts

Tibor's speech acts are triggered by S# (see Crandall et al. (2018)) as well as several other system modules. Example speech acts used by Tibor are shown in Table S1 and Table S2. This table illustrates how different speech acts can be used to communicate signals relating to Tibor's strategy, personal touch, and error recovery in different game scenarios. For example, in between rounds, S# largely communicates the feelings related to the last round of play (personal touch) as well as its intended behavior and expectations for the upcoming round (strategy). During the round, other modules (such as vision and grasping) detect anomalies which prevent normal game flow. When such anomalies are detected Tibor communicates its awareness of a particular subsystem's failure resulting in a reattempt of a task, restart of a subsystem, or requesting assistance from the human (always regardless of personal touch or strategy being enabled). Additionally, Tibor will also express feelings of frustration when failures occur and happiness upon recovery from the error (personal touch).

Facial Expressions and Gaze

In addition to verbal communication, Tibor seeks to express its feelings and consciousness through non verbal communication such as facial expressions and gaze. This was enabled only in the conditions enabling personal touch.

Tibor's facial expressions consisted of three components. The first component is a static expression reflective of Tibor's current emotional state (as communicated to it by the S# algorithm) using the faces shown in Figure S2. Five emotions are supported: neutral, joy, surprise, anger, and sadness. Secondly, to convey consciousness, Tibor blinks periodically. Finally, to give speech a life-like quality, Tibor's mouth is animated during speech.

Tibor also uses gaze, via head and eye movements, to convey its consciousness of its partner, the environment, and the game flow. For example, Tibor looks down at the user's hand as they pick up a block. Once the user has finished selecting a block, Tibor makes eye contact with its partner. Additionally, Tibor focuses its eyes on the block that it picks, and then looks at where it intends to place the block while moving it. Tibor ends its turn by both facing and looking at it's partner.

Categorizing Signals: Personal Touch vs. Strategy

The separation between communication related to strategy and communication relating to personal touch becomes blurred in some cases. We wish to be clear about how the communication was classified in our personal touch and strategic conditions.

- All non-verbal communication including expressions and gaze was attributed to personal touch.
- All verbal communication explicitly stating strategy was attributed to strategic communication.
- All verbal communication solely communicating emotion was attributed to personal touch.

- All verbal communication implicitly stating strategy, but with emotion could be classified as personal touch communication. [2]
- We attempted to not even consider communication that both explicitly stated strategy and also had emotionally charged words, as such phrases could not be considered an independent variable.

Listening

An additional aspect of S# is that it adapts its behavior based on the strategic proposals made by its partner. Tibor receives these and other speech acts from its partner via a voice interface. To account for the limited-voice recognition capabilities of our robot, we limited people to voicing the set of speech acts shown in Table S3. At the beginning of each round, S# processes all of the speech acts that the player had said since the last round. If strategic communication is enabled, Tibor responds with acceptance or rejection of any strategic proposal by the human, prior to conveying his strategy for that round.

SM 4: User Interface

Available Speech Acts

Human participants in our human-robot study were given a predefined set of speech acts they could use that the robot understood. The list of phrases are given in Table S3. The user could state any phrase they wanted at any time, though the robot only processed these messages (in batch) at the beginning of each round.

Interface

In all user studies, a scoreboard (Figure S3) was displayed to each human participant throughout the duration of the game to help them understand the state of the game. This scoreboard contained:

- A illustration of the blocks and their point values.
- A depiction of the current state of the round.
- The money earned by the player so far.
- The play history, showing the blocks selected by each player in each round.
- The chat history (For games where speech was allowed).
- Notification bar indicating whose turn it was, or whether it was time to set up the table for a new round.

Supplementary References

Bradski, G. (2000). The OpenCV Library. Dr. Dobb's Journal of Software Tools *25*.

Crandall, J. W., Oudah, M., Ishowo-Oloko, F., Abdallah, S., Bonnefon, J.-F., Cebrian, M., Shariff, A., Goodrich, M. A. and Rahwan, I. (2018). Cooperating with machines. Nature communications *9*, 233.

Gintis, H. Z. (2000). Game Theory Evolving: A Problem-Centered Introduction to Modeling Strategic Behavior. Princeton University Press.

Nash, J. F. (1950). The Bargaining Problem. Econometrica *28*, 155–162.

Nash, Jr., J. F. (1928). Zur Theorie der Gesellschaftsspiele. Mathematische Annalen *100*, 295–320.

Nash, Jr., J. F. (1950). Equilibrium points in n-person games. Proceedings of the National Academy of Sciences *36*, 48–49.

Skyrms., B. (2004). The stag hunt and the evolution of social structure. Cambridge University Press.

Sucan, I. A. and Chitta, S. (2013). MoveIt Motion Planning Framework. http://moveit.ros.org. Accessed Nov. 24 2020.

Wiedemeyer, T. (2015). IAI Kinect2. https://github.com/code-iai/iai_kinect2. Accessed June 12, 2015.

[2] To some degree, all verbal communication with emotion could be construed as having a purpose or a strategy to it. Verbal communication is often used to manipulate the actions of others or convince someone to see your side. Therefore it is hard to get rid of all 'strategy' from emotionally charged speech. However, for the purposes of this study, we decided that only explicit strategy proposals would be classified as strategic speech. In addition, we tried to minimize the amount of strategy that could be implicitly deduced from personal touch communication.
